# Improving the calling of non-invasive prenatal testing on 13-/18-/21-trisomy by support vector machine discrimination

**DOI:** 10.1371/journal.pone.0207840

**Published:** 2018-12-05

**Authors:** Jianfeng Yang, Xiaofan Ding, Weidong Zhu

**Affiliations:** 1 Guangzhou DaAn Clinical Laboratory Center, YunKang Group, Guangzhou, Guangdong, China; 2 Applied Genomic Center, Hong Kong University of Science and Technology, Clear Water Bay, SaiKung, Hong Kong Special Administrative Region, China; Tel Aviv University, ISRAEL

## Abstract

With the advance of next-generation sequencing (NGS) technologies, non-invasive prenatal testing (NIPT) has been developed and employed in fetal aneuploidy screening on 13-/18-/21-trisomies through detecting cell-free fetal DNA (cffDNA) in maternal blood. Although Z-test is widely used in NIPT NGS data analysis, there is still necessity to improve its accuracy for reducing a) false negatives and false positives, and b) the ratio of unclassified data, so as to lower the potential harm to patients as well as the induced cost of retests. Combining the multiple Z-tests with indexes of clinical signs and quality control, features were collected from the known samples and scaled for model training using support vector machine (SVM). We trained SVM models from the qualified NIPT NGS data that Z-test can discriminate and tested the performance on the data that Z-test cannot discriminate. On screenings of 13-/18-/21-trisomies, the trained SVM models achieved 100% accuracies in both internal validations and unknown sample predictions. It is shown that other machine learning (ML) models can also achieve similar high accuracy, and SVM model is most robust in this study. Moreover, four false positives and four false negatives caused by Z-test were corrected by using the SVM models. To our knowledge, this is one of the earliest studies to employ SVM in NIPT NGS data analysis. It is expected to replace Z-test in clinical practice.

## Introduction

On the basis of the discovery of cell-free fetal DNA (cffDNA) in maternal plasma and serum [1] as well as the advance of next-generation sequencing (NGS) technology [2], Non-invasive prenatal testing (NIPT) has been developed in 2008 [3, 4] and applied in clinical use recent years for fetal aneuploidy detection mainly on Down's syndrome, Edward's syndrome and Patau's syndrome, respectively corresponding to 21-trisomy(T21), 18-trisomy(T18) and 13-trisomy(T13) [3–5]. Before the application of the NGS-based NIPT, there were mainly two methods to detect 13-/18-/21-trisomies in clinical practice. One is the non-invasive serological test with high rate of false positive and false negative [6]; the other is the golden standard—the invasive amniocentesis with a rate of 1/250 inducing abortion [7]. Comparatively, NIPT is much more accurate than serological test and safer than amniocentesis. The International Society for Prenatal Diagnosis [8], the National Society of Genetic Counselors [9], the American College of Obstetricians and Gynecologists and the Society for Maternal-Fetal Medicine [10] had published committee opinions stating that such a cffDNA testing could be offered to pregnant women at high risk for fetal aneuploidy as a screening option after counseling.

Except those employing deep sequencing or array-based methods, most NIPTs were performed using the low-coverage next-generation sequencing (NGS) platforms such as Verifi [11], Materni21 [12], panorama [13] and NIFTY [14]. Similar to copy number variation analysis, the sequencing reads from a test sample were mapped and counted as depth in bins of a certain size, following by a measurement of deviation from negative control. Since the triploid fetus has 2–5% more cffDNA than diploid fetus, Z-test was frequently employed in deviation measurement [3, 15].

Statistically, Z score indicates the significance of deviation from the baseline, e.g. Z > 3 means that the test data approximates the baseline with P < 0.001 and hence is likely to be from a triploid sample. Types of Z-tests were employed in different NIPT studies, such as Chiu et al. using average of negatives as baseline [3] and Zhang et al. using internal reference as baseline [15]. However, these one-Z-test based approaches have many problems in clinical practice. First, only single Z score is insufficient to give accurate prediction on different samples due to read distribution bias among individuals. Further, fetal fraction has been proven to be crucial in trisomy determination [16]; however, it was not involved in one-Z-test based approach in NIPT NGS data analysis. These problems could result in inaccurate prediction, high cost of re-testing and delay of treatment.

**Fig 1:**
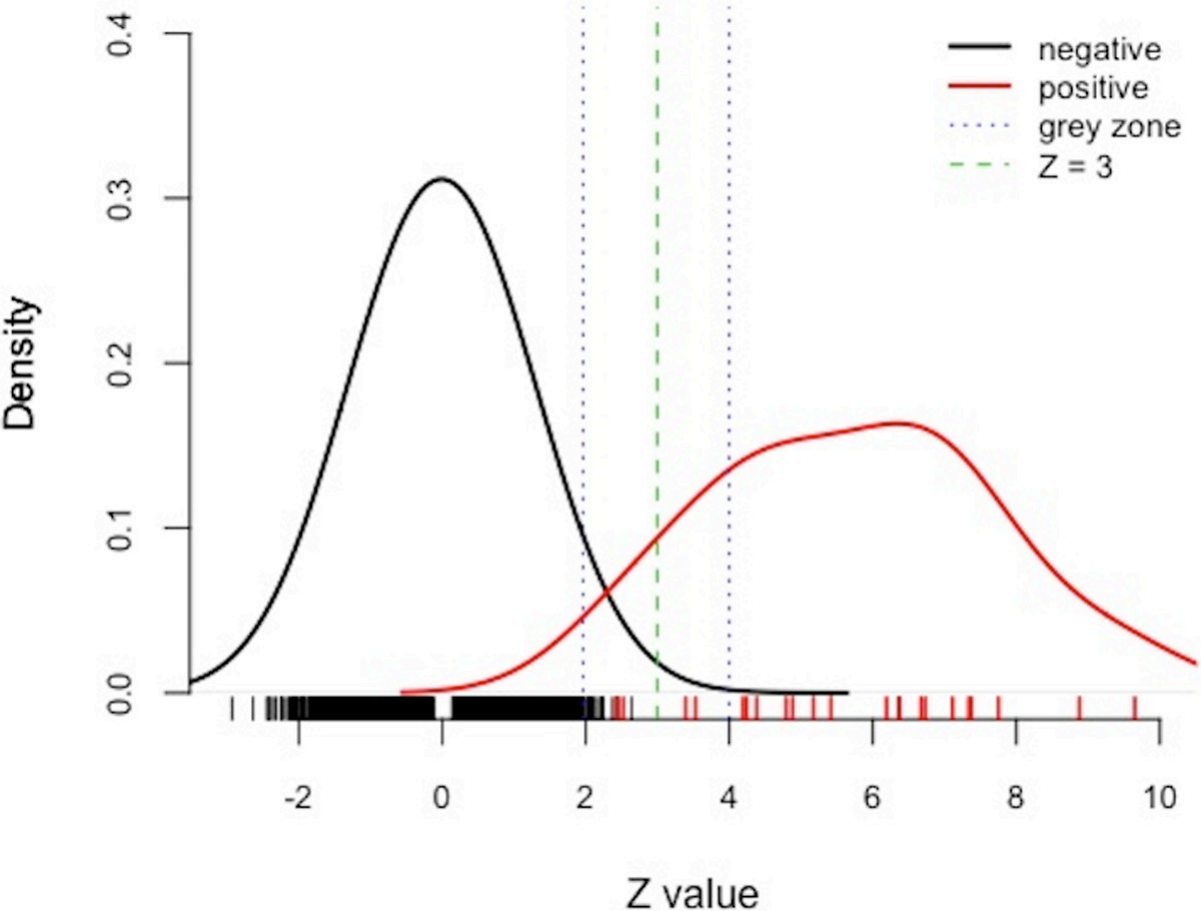
Density plot of Z scores from current one-Z-test based NIPT. Negatives and positives are shown in dark and red respectively. Green dash indicates the cutoff of Z = 3 that was frequently used as a criterion in discrimination. Blue dashes shows the “grey zone” interval between Z = 1.96 and 4, which means failure in discrimination using Z = 3, and requires a retest.

As shown in Fig 1, the distributions of Z scores of negatives and positives overlapped in a certain intervals, where the cutoff Z = 3 was unable to discriminate. A simulation shown in S1 Fig indicates that small portion of negative samples could have Z > 3 while small portion of positive samples could have Z < 3, especially when fetal fraction is around or less than 5%. In clinical practice, it is guaranteed that any sample with Z score in an interval (1.96, 4), called "grey zone", requires a retest. It is because that only using Z = 3 as cutoff to separate positives from negatives may result in inaccurate results.

Therefore, it is meaningful to develop a more precise method for NIPT data analysis. The support vector machine (SVM) is an excellent tool for this purpose. It is a supervised machine learning (ML) algorithm that identifies an arbitrarily defined framework for discriminating query data using a model build from training dataset with selected features [17]. SVM has already shown high robustness and accuracy in fields [18], such as cancer subtype classification [19], splice site prediction [20] and single nucleotide polymorphism (SNP) prediction [21].

For NIPT on 13-/18/-21-trisomies, it has been reported that positive samples are much fewer than negatives [22, 23]. Referred to a clinical experience from ~150,000 pregnancies in mainland China [23], the positive rates of 13-/18-/21-trisomies were respectively 0.045%, 0.15% and 0.52%. The large difference in number of positive and negative could lead to class imbalance in ML model training if all data were employed in research. However SVM could reduce the effect of class imbalance by selecting the support vectors from all given input data. Further, feature co-linearity would not affect the SVM model in discrimination. Therefore in this study, SVM is employed to improve the prediction on NIPT NGS data with a purpose to replace the one-Z-test based approach in current clinical practice. Combining multiple Z values with indexes of clinical signs and quality control, SVM model was trained for each dataset of 13-/18-/21-trisomy to accurately discriminate the samples, especially the "grey zone" NIPT results and those falsely predicted before.

## Materials and methods

### Specimen source

This study was a retrospective analysis on the NIPT NGS data obtained from March to July 2016 at Guangzhou DaAn Clinical Laboratory Center. Informed consent was obtained from all participants. Information such as gestational week and maternal age was obtained while the names of participants have been masked so as to protect their privacy. The trisomy samples were validated by amniocentesis. The NIPT experiments were based on the semi-conductor sequencing platform same as in Liao et al 's paper in 2013 [24]. The reported results were output through a CFDA-certified standard operation protocol (SOP) and a DaAn Gene's compiled bioinformatics plugin named "Seqboost" developed on the basis of Liao et al 's paper [24] that described a one-Z-test based approach for NIPT prediction.

### Data summary

In total 5518 NIPT data were collected during the period from two semi-conductor sequencers located in the NIPT lab in Guangzhou (Table 1). There are forty-six data from triploid samples with one data labeled "#5267" is positive in both T18 and T21 (S1 Table). Hence there are forty-seven triploid cases, respectively five for T13, fifteen for T18 and twenty-seven for T21. Average age of pregnant mother with negative results was 31.83 (95% CI: 15–51), slightly larger than the average age of ones with positive results (31.70, 95% CI: 17–47). Another 500 negative samples were recruited as reference negative control for NIPT calling.

**Table 1:** Demographic subjects of pregnant women undergoing non-invasive prenatal testing (NIPT) for aneuploidies between 1 March and 31 July in 2016.

Subject ^a^	Total	% of all	Negative	% in group	% of all	Positive ^b^	% in group	% of all	P13	% in group	% of all	P18	% in group	% of all	P21	% in group	% of all
	5518		5472		99.18			0.82	5		0.09	14		0.25	27		0.49
Age ^c^	31.83	(15–47)	31.83	(15–47)		31.70	(20–43)		31.2	(25–40)		29.57	(23–42)		32.59	(20–43)	
<24	700	12.69	691	12.63	98.71	9	20.00	1.29	0	0.00	0.00	4	28.57	0.57	5	18.52	0.71
25–29	1285	23.29	1273	23.26	99.07	12	26.67	0.93	3	60.00	0.23	5	35.71	0.39	4	14.81	0.31
30–34	1371	24.85	1368	25.00	99.78	3	6.67	0.22	0	0.00	0.00	1	7.14	0.07	3	11.11	0.22
35–40	1741	31.55	1727	31.55	99.20	14	31.11	0.80	1	20.00	0.06	1	7.14	0.06	12	44.44	0.69
>40	421	7.63	414	7.56	98.34	7	15.56	1.66	1	20.00	0.24	3	21.43	0.71	3	11.11	0.71
Week ^c^	17.19	(8–37)	17.2	(8–37)		15.93	(12–21)		13.6	(12–15)		16.21	(12–20)		16.33	(12–21)	
<13	651	11.80	643	11.75	98.77	8	17.78	1.23	2	40.00	0.31	1	7.14	0.15	5	18.52	0.77
14–27	4807	87.11	4770	87.16	99.23	37	82.22	0.77	3	60.00	0.06	13	92.86	0.27	22	81.48	0.46
>28	60	1.09	60	1.10	100.00	0	0.00	0.00	0	0.00	0.00	0	0.00	0.00	0	0.00	0.00
CostDay ^c^	10.39	(5–62)	10.38	(5–62)		10.80	(6–18)		10	(8–14)		12.07	(7–18)		10.11	(6–17)	
<7	956	17.33	947	17.30	99.06	9	20.00	0.94	0	0.00	0.00	2	14.29	0.21	7	25.93	0.73
8–14	3963	71.82	3933	71.86	99.24	30	66.67	0.76	5	100.00	0.13	9	64.29	0.23	17	62.96	0.43
15–21	561	10.17	555	10.14	98.93	6	13.33	1.07	0	0.00	0.00	3	21.43	0.53	3	11.11	0.53
>22	38	0.69	38	0.69	100.00	0	0.00	0.00	0	0.00	0.00	0	0.00	0.00	0	0.00	0.00

Demographic characteristics of pregnant women undergoing NIPT for aneuploidies in this study.

^a^ Age means the age of the pregnant mother while doing the NIPT; Week means the gestational week while doing the NIPT; CostDay means the time cost in our NIPT service.

^b^ Positive means the trisomy in either chromosome 13, 18 or 21. If none of these three chromosomes were found trisomy, the sample would be regarded as Negative in this study. P13 means trisomy in chromosome 13; P18 means trisomy in chromosome 18; P21 means trisomy in chromosome 21.

^c^ Average values of relevant subjects with minimums and maximums in the brackets.

As shown in S1 Table, a series of values were listed to demonstrate the information of these data, including "Z_run" as the Z scores output by "Seqboost" in one’s run, "Real_state" as the results confirmed by prenatal or postnatal diagnosis, fetal fraction predicted using SeqFF [25], peak value of read length, maternal age and gestational week. According to CFDA's NIPT policy and DaAn Gene's SOP, Z score = 3 is the cutoff to distinguish negatives and positives. Hence in routine NIPT, the data with "Z_run ≥3" would be regarded as positive, meaning it’s significantly deviated from the baseline of reference dataset; while those with "Z_run < 3" would be regarded as negative. Hence, the data predicted as positive with "Real_state = -1" as negative were false positives; those predicted as negative with "Real_state = 1" as positive were false negatives.

Of these 5518 data, 766 data with unique reads fewer than 3,000,000 or predicted fetal fraction less than 5% were labeled as "QC-filtered" on the basis of quality control (QC) according to the SOP. The remaining "QC-pass" 4752 data were categorized into three groups for specified chromosomes on the basis of the principle of statistics: Group "N" as those with Z scores smaller than 1.96, meaning not significantly higher than baseline of reference dataset (P > 0.05); Group "P" as those with Z scores larger than 4, meaning significantly higher than baseline of reference dataset (P < 0.0001); Group "Unclassified" as those with Z scores between 1.96 and 4, meaning retest is required for double check in nowadays' NIPT. For each specified chromosome, data in Groups "N" and "P" were employed to train models and conduct internal validation in this study. Data in Group "Unclassified" and "QC-filtered" were used in performance test. We also employed the trained model to correct the four false positives and four false negatives caused by Z-test in previous NIPT reports.

### Feature selection and data reanalysis

Reads generated from semi-conductor Ion Proton Sequencer (Life Technologies) were trimmed and mapped to human genome 19 (hg19), following by recalibration and realignment through the automated pipeline of the supporting Ion Torrent Suite Software (Life Technologies). Then reads were filtered using SAMtools' command [26] 'samtools view–F 1024 –q 10' to remove PCR duplicates and low quality (mapping quality smaller than 10) reads. Thus, the remaining high-quality unique reads were used for the following analysis. Similar with the CFDA-certified DaAn Gene's SOP, read-depth for each contiguous 20kb bin was calculated using the genomeCoverageBed program in BEDtools [27]. To remove the bias of read-depth distribution caused by data volume difference, GC content and casual sequencing bias respectively, three types of normalization were applied in four steps: 1) Intra-run normalization was used to eliminate the difference between each data; 2) Winsorization that was a transformation reducing the influence of outliers by moving observations outside a certain fractile in the distribution to that fractile [28], was employed to reduce the extreme read-depth among each contiguous window consisting of 15 bins of 20 kb; 3) LOESS was employed to remove GC-bias same as in Chiu et al. 's paper [3]; 4) Intra-run normalization again due to steps 2) and 3) could induce bias of data size. Mean and standard deviation (s.d.) of read-depth of each chromosome were calculated for further statistical analysis.

The normalized read-depth of each bin was added up every 15 bins to smooth the read-depth signal. Then the mean and standard deviation of merged read-depth on each chromosome was calculated to statistical analysis for fetal aneuploidy evaluation. For each data, six Z scores were called as described by the following formula:

$$Z\_baseline\_vs\_n_{i}=\frac{mean_{i}-mean\left(ref._{i}\right)}{s.d.\left(ref._{i}\right)}$$ (1)

where *Z_baseline_vs_n* means the Z score normalized to the average of reference negative samples on chromosome *i*, and *ref*. means the normalized read-depth values of reference negative samples.

$$Z\_baseline\_vs\_p_{i}=\frac{mean_{i}-mean\left(ref._{i}\right)\times \left(1+fetal\%/2\right)}{s.d.\left(ref._{i}\right)}$$ (2)

where *Z_baseline_vs_p* means the Z score normalized to the average of predicted reference positive data, *fetal%* means fetal DNA fraction. The predicted reference positive data is equal to the mean value of reference negative data multiplied by a factor (1+fetal%/2) based on the assumption that half of fetal fraction would be increased when trisomy happens.

$$Z\_chr\_vs\_n_{i}=\frac{mean_{i}-median\left(mean\left(sample\_chr\right)\right)}{s.d.\left(ref._{i}\right)}$$ (3)

where *Z_chr_vs_n* means the Z score normalized to the internal reference autosome value that is the median of all averages of normalized read-depth in each autosome of this sample, which was similar in Lau's paper [15].

Similarly, we have:

$$Z\_chr\_vs\_p_{i}=\frac{mean_{i}-median\left(mean\left(sample\_chr\right)\right)\times \left(1+fetal\%/2\right)}{s.d.\left(ref._{i}\right)}$$ (4)

where *Z_chr_vs_p* means the Z score normalized to the predicted positive internal reference autosome value that is the median of predicted positive averages of normalized read-depth in each autosome of this sample.

Z_sample_vs_ni=−(mean(ref.i)−meani)Sm×MADiwindowi (5)

where *Z_sample_vs_n* means the Z score normalized to the average of sample data, *MAD* means the median absolute deviation of read-depth, *window* means the number of windows on the chromosome *i*, and *S*_*m*_ is a factor equal to 1.4826 and makes Sm×MADiwindowi approximate to the standard deviation of read-depth of sample data.

Z_sample_vs_pi=−(mean(ref.i)×(1+fetal%/2)−meani)Sm×MADiwindowi (6)

where *Z_sample_vs_p*_*i*_ means the Z score normalized to the mean value of predicted positive sample data.

### SVM discrimination

Six Z scores together with fetal fraction, peak value of read length, maternal age and gestational week, were collected for support vector machine classification. For the ten features selected for SVM classification model training, the six Z score-based features were essential because their distributions between negatives and positives were significantly different (Wilcox Rank Sum test, P < 2.2×10^−16^), while the other four features were not biasedly distributed (Table 2).

**Table 2:** List of features employed in SVM classification.

Feature Number	Feature Name	Description	SVM Scale	P value ^d^
D1	Z_baseline_vs_n	Z value normalized to the baseline of control samples	No	< 2.2e^-16^
D2	Z_baseline_vs_p	Z value normalized to the baseline of predictive positive samples	No	< 2.2e^-16^
D3	Z_chr_vs_n	Z value normalized to the internal chromosome reference	No	< 2.2e^-16^
D4	Z_chr_vs_p	Z value normalized to the predictive positive internal chromosome	No	< 2.2e^-16^
D5	Z_sample_vs_n	Z value normalized to the baseline of control samples	No	< 2.2e^-16^
D6	Z_sample_vs_p	Z value normalized to the baseline of predictive positive samples	No	< 2.2e^-16^
D7	Fetal	Fetal fraction in maternal plasma	Yes	0.7542
D8	Peak	Peak value of read length distribution	Yes	0.6655
D9	MA	Maternal age	Yes	0.2541
D10	GW	Gestational week	Yes	0.5125

In total ten features were used in SVM model training and classification

^d^ Wilcoxon rank-sum test.

Ten features were collected from the data in Groups "N" and "P" for model building on specified chromosomes. The six Z scores obtained from formula (1) to (6) do not require scaling because they were already normalized, while the other 4 features including fetal fraction, peak value of read length, maternal age and gestational week, would be normalized to same scale ranging from 0 to 3 by the command 'svm-scale–l 0 –u +3'. For the training dataset, '-s' was used to save the scaling range, and '-r' was used to restore the saved scaling range on test data. Then, the SVM model was constructed by 'svm-train' and employed to do prediction by 'svm-predict' in LBSVM package. Despite SVM was quite efficient in handling sparse data, we also evaluated its performance by assigning two class weights (using '-wi' in training) that were inversely correlated with their instance number, aiming to improve the accuracy in unbalanced data.

We trained SVM models using those ten selected features from chromosomes 13, 18 and 21 datasets separately. Let us denote class labels as y_i_ ∈ {-1, 1} for normal state and trisomy of each specified chromosome, respectively. Given a set of training data {*x*_*i*_, *y*_i_}, *i* = 1, 2, …, *n*, the SVM returns a maximum margin separating hyper-plane with **w** and an offset b using

$${\text{argmin}}_{\text{w},\text{b}}\frac{1}{2}\left|{\text{w}}^{\text{T}}\text{w}\right|+\text{C}\sum_{\text{i}}\varepsilon_{\text{i}}$$ (7)

subject to: $y_{i}\left(w^{T}\phi \left(x_{i}\right)+b\right)\geq 1-\varepsilon_{i},i=1,2,\ldots ,n.$

where w are feature weights representing the hyper-plane, $\varepsilon_{i}\geq 0$are slack variables designed to allow misclassified data points, and C > 0 is the penalty parameter for misclassification.

By solving for the Lagrangian dual of formula (7), we could obtain a simplified optimization problem

$$\text{maxmize}{\sum_{x=i}^{n}\alpha }_{i}-\frac{1}{2}\sum_{i=1}^{n}\sum_{j=1}^{n}\alpha y_{i}y_{j}\alpha_{i}\alpha_{j}\phi (x_{i})\phi (x_{j})$$ (8)

subject to $\sum_{\text{x}=\text{i}}^{\text{n}}\alpha_{\text{i}}Y_{\text{i}}=0$, 0 ≤ α_i_ ≤ C for all i.

This dual problem could be efficiently solved using quadratic programming or sequential minimal optimization (SMO) algorithm.

Here, the solution for w in formula (7) is also given by

$$\text{w}=\sum_{\text{i}=1}^{\text{n}}\alpha_{\text{i}}{\text{y}}_{\text{i}}\phi \left({\text{x}}_{\text{i}}\right)$$ (9)

Once the optimal solution for α_i,…,_α_n_ is found, the optimal *b* is then determined using the maximum margin condition:

$$b=\frac{1}{2}[min_{y_{i}=1}\sum_{j=1}^{n}\alpha_{j}y_{j}\phi (x_{j})\phi (x_{i})-max_{y_{i}=-1}\sum_{j=1}^{n}\alpha_{j}y_{j}\phi (x_{j})\phi (x_{i})]$$ (10)

The decision function for any new point *x* is then

$$\text{f}=\sum_{\text{i}=1}^{\text{n}}\alpha_{\text{i}}{\text{y}}_{\text{i}}\phi \left({\text{x}}_{\text{i}}\right)\phi \left(\text{x}\right)+\text{b}$$ (11)

with *f* > 0 assign to class 1 and *f* < 0 assign to class -1.

The inner product $\phi \left(x_{i}\right)\phi \left(x_{j}\right)$ in formula (8) could also be represented as a kernel function *k*, which satisfies $k\left(x_{i},x_{j}\right)=\phi \left(x_{i}\right)\phi \left(x_{j}\right)$.

Here, we applied two kinds of kernel functions for our data: linear kernel function and radial basis function (RBF). The linear kernel function is based on inner products of input features between any two samples, so we could verify if our data are linearly separable. The feature space of the RBF kernel, on the other hand, enable us to learn a nonlinear classification by transforming input features into an implied feature space with an infinite number of dimensions.

The RBF kernel is defined as:

$$\text{k}\left({\text{x}}_{\text{i}},{\text{x}}_{\text{j}}\right)=\text{exp}\left(-{\gamma ||x}_{\text{i}}-{}_{\text{j}}\text{x||}{}^{2}\right)$$ (12)

where γ is a kernel parameter controlling the sensitivity of the kernel function. For trainings with linear kernel function, only one hyper-parameter C needs to be adjusted to select an appropriate model through k-fold cross validation. A low C makes the decision boundary smooth, while a high C could select more samples as support vectors and classify more training samples correctly, which thus is prone to be over-fitting. For the RBF kernel, besides C, there is another kernel parameter γ, defining the extent of influence for those support vectors, with high values meaning a narrow range of influence. To select optimal parameters (C and γ for RBF kernel; C for linear kernel), we employed a grid search approach with 0.1 as step and 5-fold cross validation by using grid.py from LIBSVM [17] and expand.grid from 'caret' package [29]. And to prevent over-fitting, C values were carefully checked to avoid solutions with large values.

### Other discrimination methods

Other discrimination methods such as linear discriminant analysis (LDA) [28], quadratic discriminant analysis (QDA), decision tree (Dtree) were also tested on the same NIPT dataset in this study.

Both of LDA and QDA assumed samples drawn from a multivariate normal distribution N(μ,Σ) with mean vector μ and covariance matrix Σ. The probability of k class was given by:

$$P\left(k|x\right)=\frac{\pi_{k}P(x|k)}{P\left(x\right)}$$ (13)

where $\pi_{k}$ was the prior class probability of *k* classes.

LDA arises when the covariance matrix for each classes were assumed to be the same, in which case the discrimination boundary could be simplified to:

$$\delta \left(x\right)=x^{T}\Sigma^{-1}\mu_{k}-\frac{1}{2}\mu_{k}^{T}\Sigma^{-1}\mu_{k}+log\pi_{k}$$ (14)

In QDA analysis, the covariance matrix from each class is different and its discrimination boundary is:

$$\delta \left(x\right)=-\frac{1}{2}|\Sigma_{k}{}^{-1}|-\frac{1}{2}{\left(x-\mu_{k}\right)}^{T}\Sigma_{k}{}^{-1}\left(x-\mu_{k}\right)+log\pi_{k}$$ (15)

where $\Sigma_{k}$ was the covariance matrix for k class; LDA and QDA were trained by the 'lda' and 'qda' methods in 'MASS' package, respectively.

For Dtree, we directly applied the 'ctree' method in the R package 'party', which utilized a binary recursive portion approach to rank and select those input variables according to their association with the input classes. We also employed AdaBoost to create a highly accurate prediction rule using the 'caret' package [29]. We implemented AdaBoost.M1 with decision trees as weak learners. The final classifier of AdaBoost was a weighted combination of weak classifiers,

$$h_{fin\left(x\right)}=argmax_{y\in Y}\sum_{t:h_{t}\left(x\right)=y}\text{ln}\left(\frac{1}{\beta_{t}}\right)$$ (16)

Where h_t_, β_t_ were the induced weak classifiers and their assigned weights, respectively. The AdaBoost model was also trained with the same input as SVM and 5-fold cross validation to avoid the chance of over fitting.

### Performance tests

The performances of SVM models trained using different hyper-parameter settings were compared respectively for chromosomes 13/18/21. Generally, four types of SVM models were compared: RBF kernel without class weight, RBF kernel with class weight, linear kernel without class weight and linear kernel with class weight. Firstly for the data in Groups "N" and "P" on specified chromosomes, an internal validation was done using the model built based on these data themselves. Importantly, the trained models were applied to predict the data in Group "Unclassified", which was the most meaningful application in this study. As well, the models were applied to predict the data in Group "QC-filtered".

Similarly, we tested the SVM models trained using different parameter settings. Performances of models trained using two kernel functions were compared. Also, model trained using class weight was compared to those trained without using class weight.

Secondly, other ML models such as LDA, QDA and Dtree, were tested in prediction of 13-/18-/21-trisomies on the three groups of datasets. The performances of other ML models were compared with the performances of SVM models mentioned above. We also conducted a comparison between SVM models with optimal parameters and Adaboost.

For visualization of performances of ML models, four ML models trained using feature D1 from formula (1) and feature D3 from formula (3) were tested in internal validation of chromosome 21. The two-dimension hyper-planes for discrimination were plotted using 'contour' in R package 'graphic'. We also visualized the performance of SVM models using three features (features D1, D3 and D7 as fetal fraction) in a 3-D plot and corresponding three 2-D plots.

## Results

### Inaccuracy of one-Z-test approach in NIPT prediction

**Fig 2:**
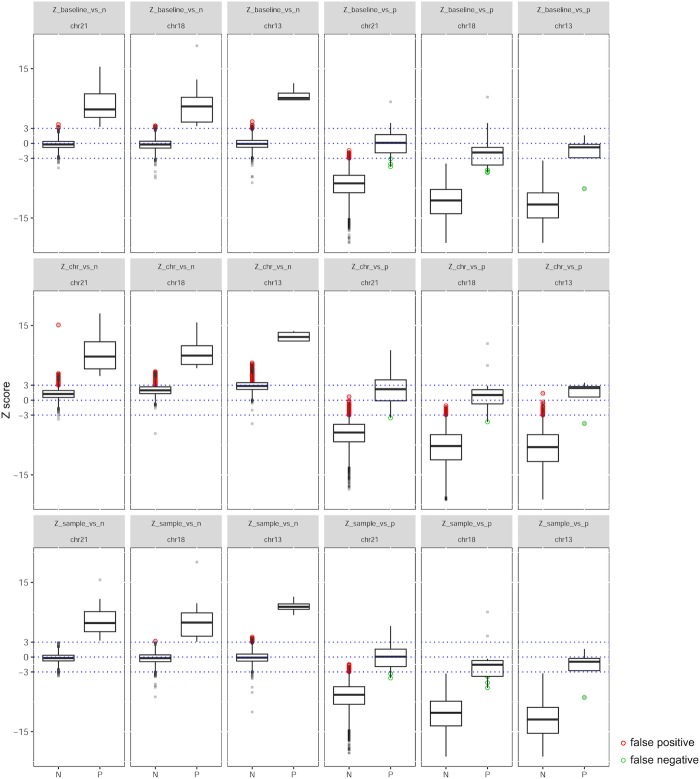
Boxplots of Z scores from six types of Z tests on chromosomes 13/18/21. The six types of Z tests were corresponding with formulas (1) to (6) in Methods. "N" means negatives and "P" means positives. Red dots represent false positives and green dots represent false negatives.

We employed all of six Z-tests from formulas (1) to (6) to demonstrate their distributions of Z scores on respectively chromosomes 13/18/21 in all QC-pass NIPT NGS data. As shown in Fig 2, none of Z-test could clearly distinguish positives from negatives. Using Z = 3 as cutoff, Z scores from formulas (1), (3) and (5) were able to identify all the true positives but a number of false positives existed especially formula (3). Using Z = -3 as cutoff, Z scores from formulas (2), (4) and (6) had both false positives and false negatives in discrimination. Though these six Z scores were significantly biased in distributions between positives and negatives (Table 2, Wilcox Rank Sum test, P < 2.2×10^−16^), the simple discriminating method that was based on a certain cutoff value would always give false positive or negatives in NIPT calling. Except these six Z scores, the other four indexes were not significantly biased in distribution between positives and negatives (Table 2).

This result suggests that the one-Z-test based approach could not guarantee the prediction accuracy of NIPT NGS data because of the simplicity of discriminating rule. Hence the calling of NIPT demands a more comprehensive approach that could combine different vectors to improve the accuracy.

### Performance of SVM models

**Fig 3:**
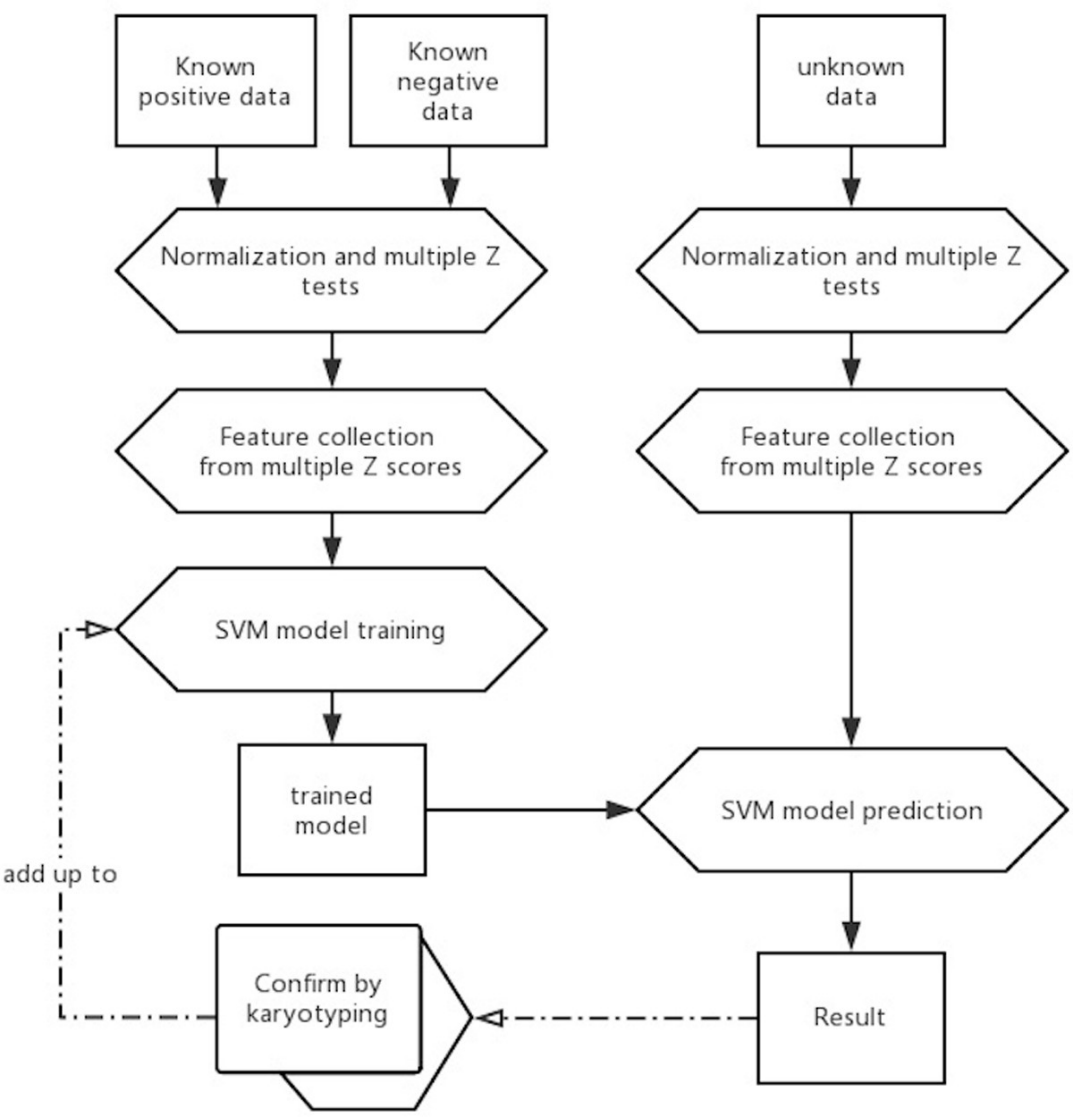
Strategy of employing SVM models to improve NIPT calling. The SVM models were trained using known datasets. Once after confirmation, validated data could be added up to the training dataset to enhance the prediction.

As described in the pipeline (Fig 3), we trained the SVM models using the known datasets from Group "N" and "P" for chromosomes 13/18/21 respectively. Firstly, models were respectively trained using different hyper-parameter settings for chromosomes 13/18/21. To compare the performances between kernel functions, both linear and RBF kernel functions were employed to build the models. Further, parameter '-wi' was used to adjust C for class weight. Parameter optimization was employed to find the best C and γ using a grid search method with 0.1 as step and 5-fold cross-validation (See Methods).

On one hand, the models with using RBF kernel performed better than the ones with linear kernel (Table 3). The models with RBF kernel achieved 100% accuracy in both internal (Group "N" & "P") and external validation (Group "Unclassified") for all three specified chromosomes. However, the models with linear kernel did not predict the positives in Group "Unclassified" well. On the other hand, the models using class weight performed as good as those not using. It was because that the models not using class weight were sufficiently accurate in the datasets of this study (S2 Table). Taken together, the SVM models with using RBF kernel function and class weight were selected in the following analysis.

**Table 3:** Performance of SVM models on NIPT prediction using different parameter setting.

Chr21			Group "N" & "P"				Group "Unclassified"			
Model ^e^	Real status	Support vector number	Prediction		Sens. ^f^	Spec.	Prediction		Sens.	Spec.
			N	P			N	P		
SVM-RBF-opt	N	365	4672	0	100.00%	100.00%	57	0	100.00%	100.00%
	P	19	0	19			0	4		
SVM-linear-opt	N	2	4672	0	100.00%	100.00%	57	0	0.00%	100.00%
	P	2	0	19			4	0		
SVM-RBF-opt-w	N	478	4672	0	100.00%	100.00%	57	0	100.00%	100.00%
	P	19	0	19			0	4		
SVM-linear-opt-w	N	2	4672	0	100.00%	100.00%	57	0	0.00%	100.00%
	P	2	0	19			4	0		
Chr18			Group "N" & "P"				Group "Unclassified"			
Model	Real status	Support vector number	Prediction		Sens.	Spec.	Prediction		Sens.	Spec.
			N	P			N	P		
SVM-RBF-opt	N	106	4697	0	100.00%	100.00%	44	0	100.00%	100.00%
	P	7	0	7			0	4		
SVM-linear-opt	N	2	4697	0	85.71%	100.00%	44	0	0.00%	100.00%
	P	2	1	6			4	0		
SVM-RBF-opt-w	N	303	4697	0	100.00%	100.00%	44	0	100.00%	100.00%
	P	6	0	7			0	4		
SVM-linear-opt-w	N	3	4697	0	85.71.00%	100.00%	44	0	0.00%	100.00%
	P	1	1	6			4	0		
Chr13			Group "N" & "P"				Group "Unclassified"			
Model	Real status	Support vector number	Prediction		Sens.	Spec.	Prediction		Sens.	Spec.
			N	P			N	P		
SVM-RBF-opt	N	1976	4706	0	100.00%	100.00%	42	0	NA	100.00%
	P	4	0	4			0	0		
SVM-linear-opt	N	2	4706	0	100.00%	100.00%	42	0	NA	100.00%
	P	2	0	4			0	0		
SVM-RBF-opt-w	N	2070	4706	0	100.00%	100.00%	42	0	NA	100.00%
	P	4	0	4			0	0		
SVM-linear-opt-w	N	2	4706	0	100.00%	100.00%	42	0	NA	100.00%
	P	2	0	4			0	0		

Four types of SVM models were compared in both internal and external validation for each of chromosome 13/18/21.

^e^ w means employing class weight to adjust parameter C; opt means employing optimization for parameters C and gamma in cross validation.

^f^ Sens. is short for sensitivity; Spec. is short for specificity.

In internal validations, the SVM models predicted the training dataset with 100% accuracies on all three chromosomes. For chromosome 21, 4691 data were employed in model training, including 19 positives (Z score > 4) and 4672 negatives (Z score < 1.96). Of these 4691 data, 497 were effective as support vectors in model training, including 19 positives and 478 negatives. For chromosome 18, 4704 data were employed in model training, including 7 positives and 4697 negatives. Of these 4704 data, 309 were effective as support vectors in model training, including 6 positives and 303 negatives. For chromosome 13, 4710 data were employed in model training, including 4 positives and 4706 negatives. Of these 4710 data, 2074 were effective as support vectors in model training, including 4 positives and 2070 negatives.

Importantly, as in external validation, the SVM models performed good in predicting the QC-pass data that used to be predicted as "Unclassified" (1.96 ≤ Z score ≤ 4) by Z-test. For chromosome 21, all 61 data in "grey zone" (1.3% of all QC-pass data) were accurately predicted using the training model, including four positives and fifty-seven negatives. It was noted that two of the four positives having Z score smaller than 3 (2.44 and 2.52 respectively), however, the SVM model could correct these false negatives. This result indicated that false negatives would be induced if only using Z score = 3 as discrimination cutoff that was employed by previous studies [3, 24]. Fortunately, SVM model trained by known dataset could uncover such false negatives. For chromosome 18, all 48 data in "grey zone" (1.0% of all QC-pass data) were accurately predicted using the trained SVM model, including 4 positives and 44 negatives. For chromosome 13, all 42 data in "grey zone" (0.9% of all QC-pass data) were accurately predicted as "negative". In summary, all of the data that could not be classified using Z-test (nearly 3% of all QC-pass data), were precisely predicted using the corresponding trained SVM models. This result suggested that SVM model could save around 3% of resource in retests. In consideration of millions of NIPTs were taken each year, such a reduction of cost is meaningful in clinical practice.

Surprisingly, the SVM models also acted effectively in predicting the 766 QC-filtered data (S2 Table). For chromosome 21, the model precisely predicted all the QC-filtered data, including 4 positives and 762 negatives. For chromosome 18, 763 out of 766 data were correct (99.61%). One positive was wrongly predicted as "negative" with prediction probability 64%, while two negatives were incorrectly predicted as "positive" with predicted probabilities 88.6% and 97.6% respectively. For chromosome 13, 765 out of 766 data were correct (99.87%). Only one positive data that was regarded as "negative" with Z score = 2.79, was also incorrectly predicted as "negative" by the SVM model. This demonstrated that the SVM model could perform well in most of QC-filtered data but could not uncover all false negatives, suggesting that quality control is still necessary to guarantee the accuracy of NIPT.

### Comparison with other ML models

Compared with other ML models, SVM models performed relatively better in the datasets of this study. SVM models obtained 100% accuracy in both internal validation and prediction on "Unclassified" dataset (Table 4). For internal validation, LDA model showed three false negatives in chromosome 21 and one in chromosome 18, QDA model showed three false positives in chromosome 21 and seven false negatives in chromosome 18, and Dtree model showed three false positives in chromosome 13. For the prediction on "Unclassified" dataset, LDA model showed only 25% sensitivity in chromosome 21 and 50% sensitivity in chromosome 18, QDA model had one false negative and four false positives in chromosome 21 and could not predict any positive in chromosome 18, and Dtree model had high rate of false positives in all three chromosomes. We also compared Adaboost that employed Dtree in model training with SVM models in prediction on training dataset (S2 Fig). Both SVM models using different kernels have similar high accuracy with Adaboost in chromosome 21 and 18, while SVM models performed slightly better in chromosome 13. This result may indicate that SVM models performed comparatively well in NIPT prediction under this dataset. However, other ML models also have the potentials in improving calling. Like Adaboost employing Dtree to create a high accurate prediction rule can enhance the accuracy. Similarly, neuronal network could also employ a class of model to create a high accurate prediction rule.

**Table 4:** Performance of different discrimination models on NIPT prediction using ten selected features.

Chr21		Group "N" & "P"				Group "Unclassified"			
Model ^g^	Real status	Prediction		Sens. ^h^	Spec.	Prediction		Sens.	Spec.
		N	P			N	P		
**SVM**	**N**	**4672**	**0**	**100.00%**	**100.00%**	**57**	**0**	**100.00%**	**100.00%**
	**P**	**0**	**19**			**0**	**4**		
LDA	N	4672	0	84.20%	100.00%	57	0	25.00%	100.00%
	P	3	16			3	1		
QDA	N	4669	3	100.00%	99.90%	53	4	75.00%	93.00%
	P	0	19			1	3		
Dtree	N	4672	0	100.00%	100.00%	51	6	100.00%	89.50%
	P	0	19			0	4		
Chr18		Group "N" & "P"				Group "Unclassified"			
Model	Real status	Prediction		Sens.	Spec.	Prediction		Sens.	Spec.
		N	P			N	P		
**SVM**	**N**	**4697**	**0**	**100.00%**	**100.00%**	**44**	**0**	**100.00%**	**100.00%**
	**P**	**0**	**7**			**0**	**4**		
LDA	N	4697	0	85.70%	100.00%	44	0	50.00%	100.00%
	P	1	6			2	2		
QDA	N	4697	0	0.00%	100.00%	44	0	0.00%	100.00%
	P	7	0			4	0		
Dtree	N	4697	0	100.00%	100.00%	40	4	100.00%	90.90%
	P	0	7			0	4		
Chr13		Group "N" & "P"				Group "Unclassified"			
Model	Real status	Prediction		Sens.	Spec.	Prediction		Sens.	Spec.
		N	P			N	P		
**SVM**	**N**	**4706**	**0**	**100.00%**	**100.00%**	**42**	**0**	**NA**	**100.00%**
	**P**	**0**	**4**			**0**	**0**		
LDA	N	4706	0	100.00%	100.00%	42	0	NA	100.00%
	P	0	4			0	0		
QDA	N	4706	0	100.00%	100.00%	42	0	NA	100.00%
	P	0	4			0	0		
Dtree	N	4703	3	100.00%	99.90%	25	17	NA	59.50%
	P	0	4			0	0		

Four types of ML models were compared in both internal and external validation for each of chromosome 13/18/21.

^g^ Corresponding R packages were employed to build models for each ML algorithms except SVM that employed libSVM. It is because libSVM is comparably applicable in parameter selection. Here SVM is default parameter setting; LDA means linear discriminant analysis; QDA means quadratic discriminant analysis; Dtree means decision tree.

^h^ Sens. is short for sensitivity; Spec. is short for specificity; and Accu. is short for accuracy.

**Fig 4:**
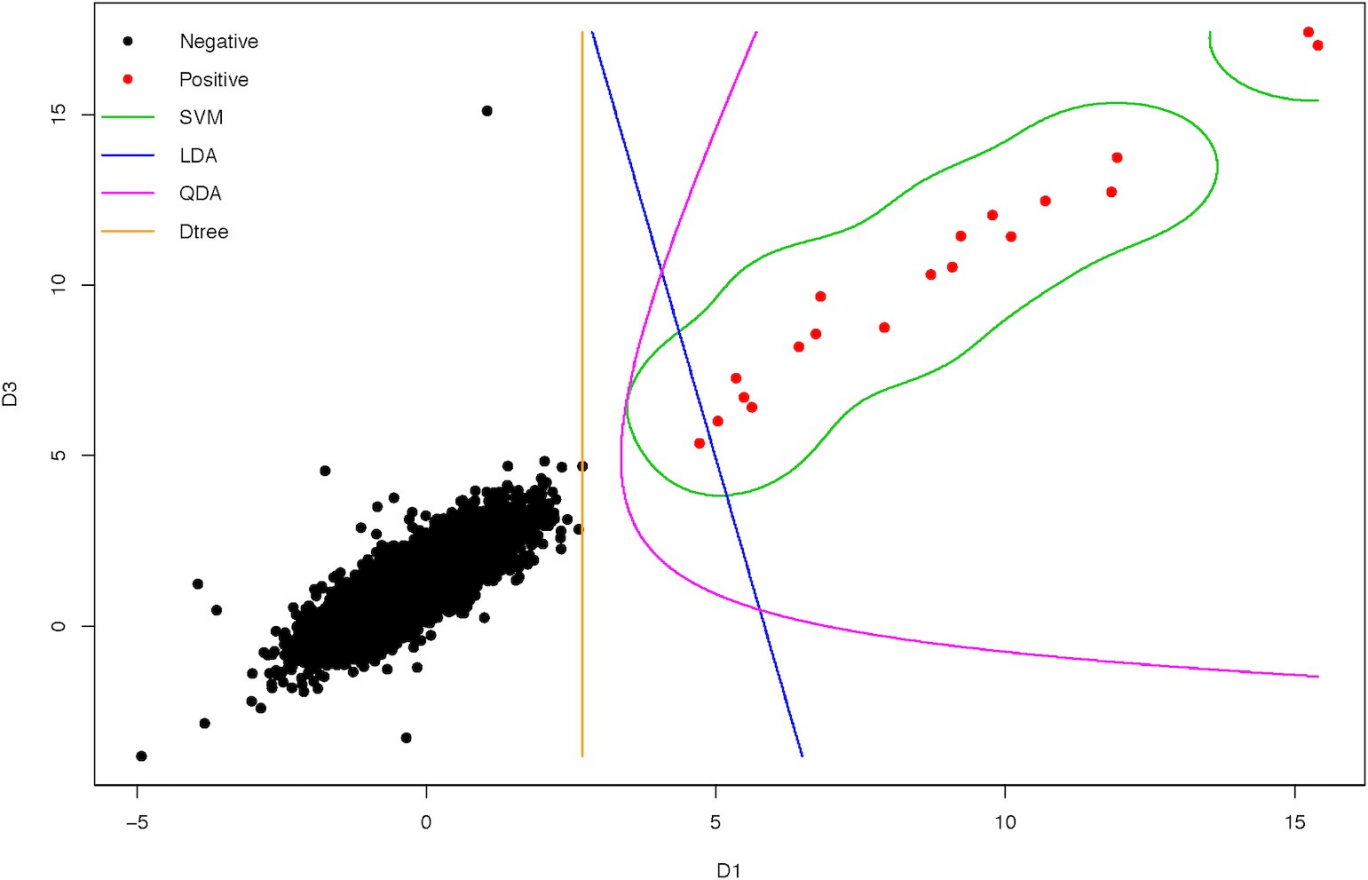
A 2-D contour plot of four ML models on NIPT data of Groups "N" and "P" on chromosome 21. Features D1 and D3 were applied in this visualization and represented as X-axis and Y-axis respectively. Dark solid points illustrate the negative samples and red solid points the positive samples. The four two-dimension hyper-planes for discrimination (green for SVM, blue for LDA, pink for QDA and orange for decision tree) were drawn on the basis of predicted categories, using 'contour' in R package 'graphic'.

We took chromosome 21 as an example to visualize how the SVM models worked (Fig 4). Using two out of ten features (D1 from formula (1) and D3 from formula (3)), nearly all of four ML models illustrated good discrimination lines on the training dataset (Groups "N" and "P"), except that LDA has one false negative. A 3-D plot and its three 2-D plots were also given to show how SVM model works in discriminating negatives and positives (S3 Fig), using feature D1, D3, and D7 (fetal fraction). These results were for visualization of how the ML models discriminated the data, whereas all of ten features were used in model training.

### Correcting the previous false callings caused by Z-test

We also employed the trained SVM models to predict eight cases that were wrongly predicted by Z-test before. As shown in Table 5, all of eight samples were corrected by using the SVM classification model, according to the prediction probabilities. For the four false positives reported previously, values of features D1, D3 and D5 (from formulas (1), (3) and (5), see Methods) significantly exceeded 3, while values of features D2, D4 and D6 (from formulas (2), (4) and (6)) also significantly lower than -3. Similarly, four false negatives also showed ambiguous values of features D1 to D6, suggesting that none of these six Z scores were reliable to do prediction independently. This further demonstrated that the SVM model was better than the commonly used one-Z-test based approach.

**Table 5:** Correction of previous false negatives and false positives by current SVM model.

Sample	Error type ^i^	Reported Z score	D1 ^j^	D2	D3	D4	D5	D6	D7	D8	D9	D10	Probability of negative in SVM prediction	Probability of positive in SVM prediction
chr13														
13_FP_1	FP	3.61	7.80908	-18.4017	10.4249	-15.4931	8.12209	-19.1393	22.38411066	151.515	32	17	0.998877	0.00112284
13_FP_2	FP	4.78	7.74425	-9.47269	9.7306	-7.34031	8.2322	-10.0696	14.70334619	130.36	25	17	0.998162	0.00183793
chr18														
18_FN_1	FN	1.77	3.31662	-2.6893	5.82699	-0.108174	3.20528	-2.59902	5.63756896	119.412	42	17	1.71E-007	1
18_FN_2	FN	1.43	5.54394	-3.20759	8.52815	-0.100806	5.79965	-3.35554	8.214781802	149.353	41	16	1.00E-007	1
18_FP_1	FP	3.22	3.36731	-10.1558	4.56993	-8.8769	3.31148	-9.98747	12.69375338	153.482	26	17	0.984857	0.015143
18_FN_3	FN	1.93	5.92865	-4.21056	6.68878	-3.41427	4.61288	-3.2761	15.25077553	148.944	27	18	2.13E-005	0.999979
chr21														
21_FP_1	FP	2.27	2.17527	-10.4262	4.38166	-8.02835	2.35229	-11.2747	17.3586114	139.857	25	17	0.997866	0.00213356
21_FN_1	FN	1.76	6.7041	-3.30991	8.41899	-1.47674	5.21281	-2.57365	13.79432935	150.056	36	12	0.00435514	0.995645

The SVM model trained in this study corrected four false negatives and four false positives previously called by one-Z-test method.

^i^ FP means false positive and FN means false negative.

^j^ The definitions of features D1 to D10 were given in Table 2.

In summary, SVM model has shown its potential in discrimination of NIPT results in this study, especially compared with the current one-Z-test based method. The SVM models using RBF kernel achieved 100% accuracies in trisomy detection of 13/18/21 in both internal and external validation. With this improvement, it is expected to reduce the cost of retests on samples in grey zone as well as the cost caused by false positives and false negatives. As shown in Fig 3, we expect that the SVM model could be further improved if 1) more known data were validated and added up to model-training; 2) more impacted features were discovered and added up to model-training. Some other clinical signs such as the values from serological test could be employed together with NIPT data to do prediction. In future, we plan to validate and optimize our ML method for trisomy prediction in a larger dataset.

## Discussion

Due to the inaccuracy of serological testing and the potential harm of amniocentesis, NIPT is suggested to be adopted in nowadays' prenatal screening with purpose to detect 13-/18-/21-trisomies. In clinical practice, positive result of NIPT would be suggest to take amniocentesis while negative result does not require. Therefore, a false negative NIPT report would result in wrong diagnosis and delay of treatment, while a false positive NIPT report would require the patient to take an unnecessary amniocentesis with a risk of abortion. Some may argue that the current one-Z-test based NIPT prediction approach is precise enough, however, let us do a simple calculation: assume that 1,000,000 women come take NIPT and 1% of them have trisomy fetuses, which means there are 990,000 negatives and 10,000 positives; based on the accuracy of one-Z-test approach provided by Chiu et al. [3] (Sensitivity 97.9% and Specificity 99.7%), there would be 210 false negatives and 2970 false positives; this means 210 women would be wrongly diagnosed and give birth to trisomy fetuses and 2970 women would take an unnecessary amniocentesis with 12 of them would miscarry the normal fetus when taking amniocentesis. In fact, the number of newborn in China was 4 million in 2015 [30] but it is believed to increase in future since the implementation of two-child policy. Therefore we are motivated to improve the accuracy of prediction on NIPT NGS data, which the one-Z-test based approach could not satisfy.

Recently, many groups have noted that the one-Z-test approach cannot satisfy the accurate prediction on NIPT NGS data. Bayindir et al. supplemented a Meta Z-test, which means the Z test of Z score, in discrimination [31]. Yu et al. improved the count-based analysis by supplementing another size-based approach [32]. Using more cutoff values is a good way to ensure the prediction accuracy of positives and negatives, however it would also increase the number of unclassified samples and hence demand more retests. Further, the fetal fraction was a key factor that could influence the prediction of one-Z-test approach, however it was not considered in one-Z-test approaches like Chiu et al. 's [3], Chen et al. 's [5] and Liao et al. 's [24]. BGI’s NIFTY employed a logarithmic likelihood odds ratio between binary hypotheses that took fetal fraction in consideration [14], but it still relies on a single-dimension cutoff to predict the result. Other information like maternal age is also important in NIPT NGS data prediction [33]. In this study we showed that ML method is a good way to solve the problems above. Combining multiple Z-tests and other features, the trained SVM models achieve extremely high prediction accuracy and decrease the number of unclassified data. In fact the enhancement is instantaneous as there is few steps in parameter optimizing. Both linear and RBF kernels can achieve same high accuracy in prediction. This suggests that positives and negatives have significant differences in the distributions of selected features.

We also noted that other ML methods could achieve similar improvement. Since the effectiveness of ML depends on the selected features and dataset, it is uncertain that SVM definitely performs best in NIPT NGS data analysis. However we have achieved our goal of improving the NIPT prediction accuracy to an extremely high level by using SVM models. We also tested some other ML methods using the same features in this study. For LDA and QDA, collinearity between features could be one of reasons of lower accuracy in prediction, while SVM allows collinearity between features. Besides SVM, Adaboost also had high accuracy in prediction. It is worth to keep testing these machine-learning algorithms if there are more features and more data in future, since our objective is to find the best approach for clinical use. Temporarily, SVM showed the most robustness according to this study. For the ten features selected for current SVM model training, the four non-Z-score features actually were not significantly biased in distributions between negatives and positives, though IONA’s paper reported that maternal age was useful in correcting its NIPT results [33], which might be due to the differences in sample composition.

In conclusion, we developed an accurate SVM-based approach and showed its potential in trisomy prediction on chromosomes 13/18/21. Compared with the one-Z-test approach, it has advantages in prediction accuracy and effectiveness, resulting in lower rate of false result and lower cost of retest. Other MLs could also improve the prediction accuracy on NIPT NGS data, and SVM is suggested according to this study. Such a ML approach could also have potential in detection of aneuploidy of other chromosomes or even micro-duplication and deletion, which would be included in our program if sufficient diagnosed cases were available. For further validating and optimizing our ML methods, we are planning to gather a larger and more comprehensive dataset in future. We suggest that the ML methods would be employed in NIPT prediction instead of the one-Z-test based approach in clinical practice.

## Supporting information

S1 Fig Z value distributions of current one-Z-test based NIPT in simulation. Each of the three normal distributions were simulated by bootstrapping 10,000 times for negative samples (green line), positive samples with fetal fraction 5% (cyan line) and positive samples with fetal fraction 10% (red line) respectively. Yellow dash line means Z score equal to 3. Dark dash lines show the interval of grey zone. When fetal DNA fraction is around 5% that is possible to happen in real, it became difficult to distinguish positives and negatives from samples in grey zone.
(TIFF)

S2 Fig Comparison of 5-fold cross validation among two SVM models and Adaboost model. (A) Chromosome 21; (B) Chromosome 18; (C) Chromosome 13.
(TIFF)

S3 Fig A 3-D contour plot and its relevant 2-D contour plots on NIPT data of Group "N" and "P" on chromosome 21. Features D1, D3 and D7 were employed in this visualization and represented as X-axis, Y-axis and Z-axis respectively. Dark solid points illustrate the negative samples and red solid points the positive samples.
(TIFF)

S1 Table Information of the 5,518 samples employed in this study. Each sheet represents the relevant information of all samples in each of chromosome 13/18/21. A series of values are listed for each of chromosome 13/18/21, including the demographic subjects, six types of Z scores obtained from formulas (1) to (6), real actual result and grouping information.
(XLSX)

S2 Table Detailed information of performance test of SVM models on NIPT prediction using different parameter setting. Column of Group "N"&"P" is the result of internal validation; column of Group "Unclassified" is the result of external validation for the QC-pass data that could not be classified by one-Z-test method; column of Group "QC-filtered" is the result of external validation for the QC-filtered data. The rows of SVM models using RBF kernel with class weight and optimal parameter setting are bold.
(XLSX)

S3 Table Detailed information of performance test of different ML models on NIPT prediction. Column of Group "N"&"P" is the result of internal validation; column of Group "Unclassified" is the result of external validation for the QC-pass data that could not be classified by one-Z-test method; column of Group "QC-filtered" is the result of external validation for the QC-filtered data. The rows of SVM models are bold.
(XLSX)

## Abbreviations

NGS: Next-Generation Sequencing
NIPT: Non-Invasive Prenatal Testing
SVM: Support Vector Machine
RBF: Radical-Based Function
CFDA: Chinese Food and Drug Administration
DNA: Deoxyribonucleic Acid
LDA: Linear Discriminant Analysis
QDA: Quadratic Discriminant Analysis
QC: Quality Control
