# Supplementary figures and images for: Improving the calling of non-invasive prenatal testing on 13-/18-/21-trisomy by support vector machine discrimination
Source: PLoS One. 2018 Dec 5;13(12):e0207840. doi: 10.1371/journal.pone.0207840 (PMC6281214; doi:10.1371/journal.pone.0207840)

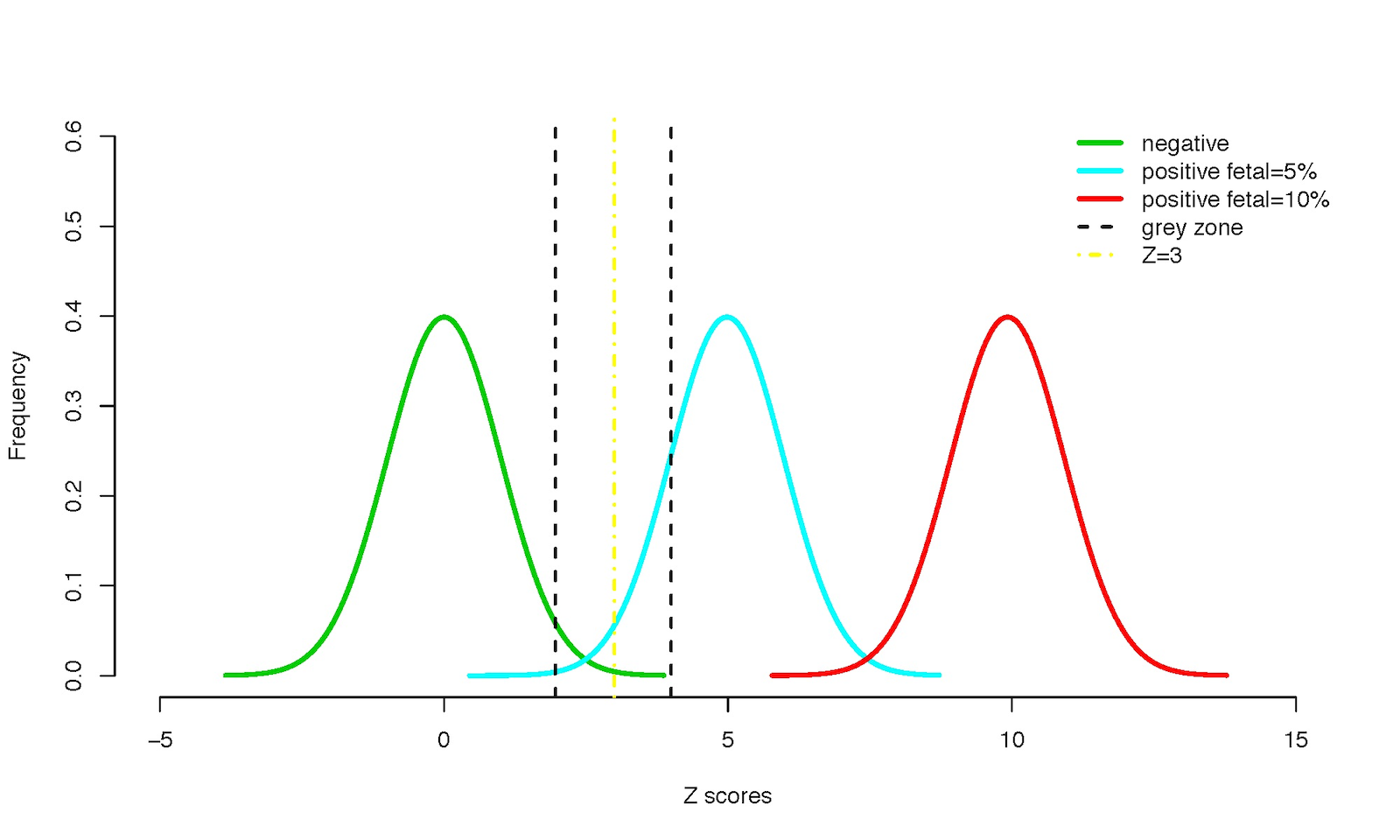

Supplement: S1 Fig — Each of the three normal distributions were simulated by bootstrapping 10,000 times for negative samples (green line), positive samples with fetal fraction 5% (cyan line) and positive samples with fetal fraction 10% (red line) respectively. Yellow dash line means Z score equal to 3. Dark dash lines show the interval of grey zone. When fetal DNA fraction is around 5% that is possible to happen in real, it became difficult to distinguish positives and negatives from samples in grey zone. (TIFF) [file pone.0207840.s001.tiff]

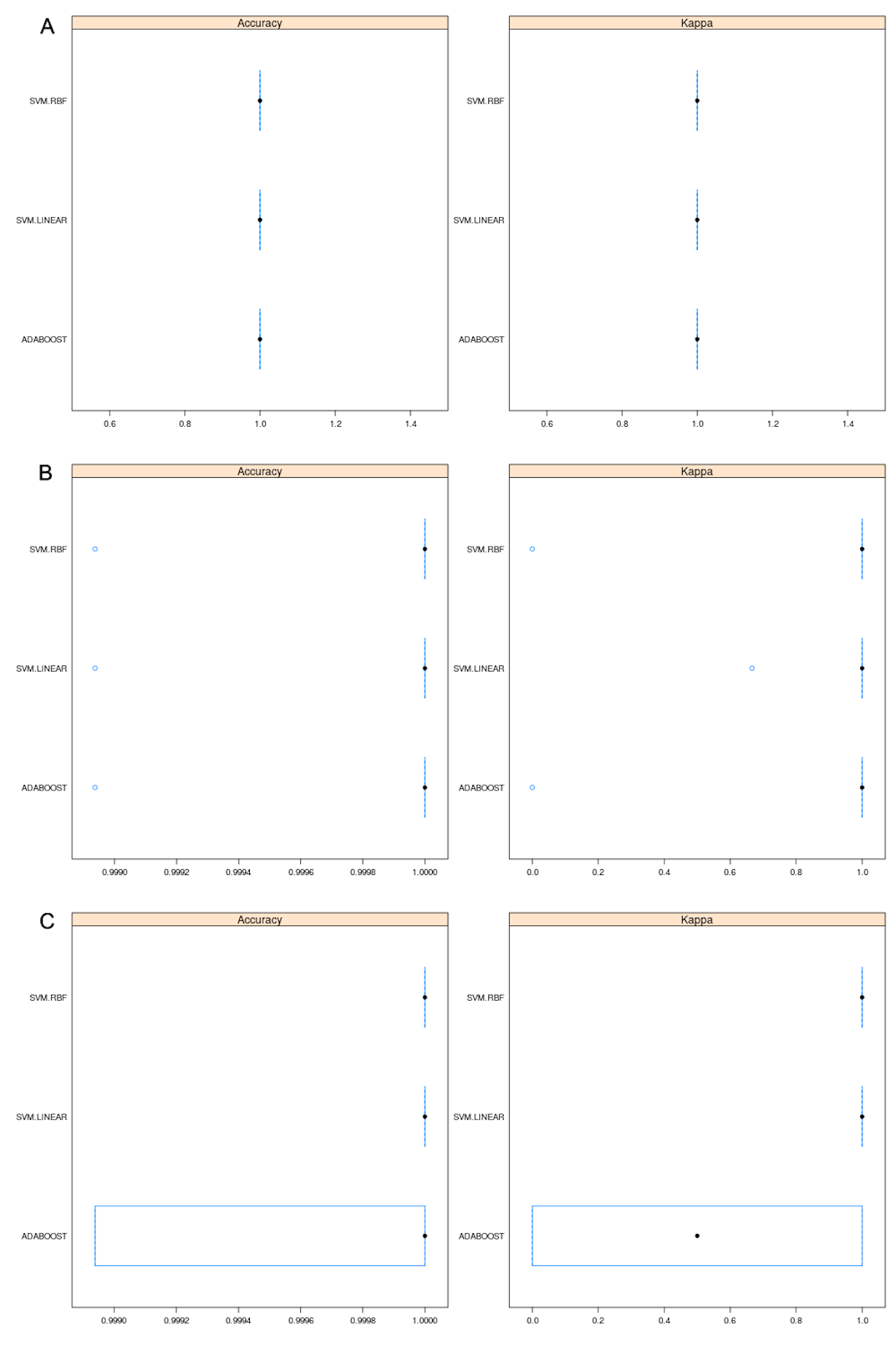

Supplement: S2 Fig — (A) Chromosome 21; (B) Chromosome 18; (C) Chromosome 13. (TIFF) [file pone.0207840.s002.tiff]

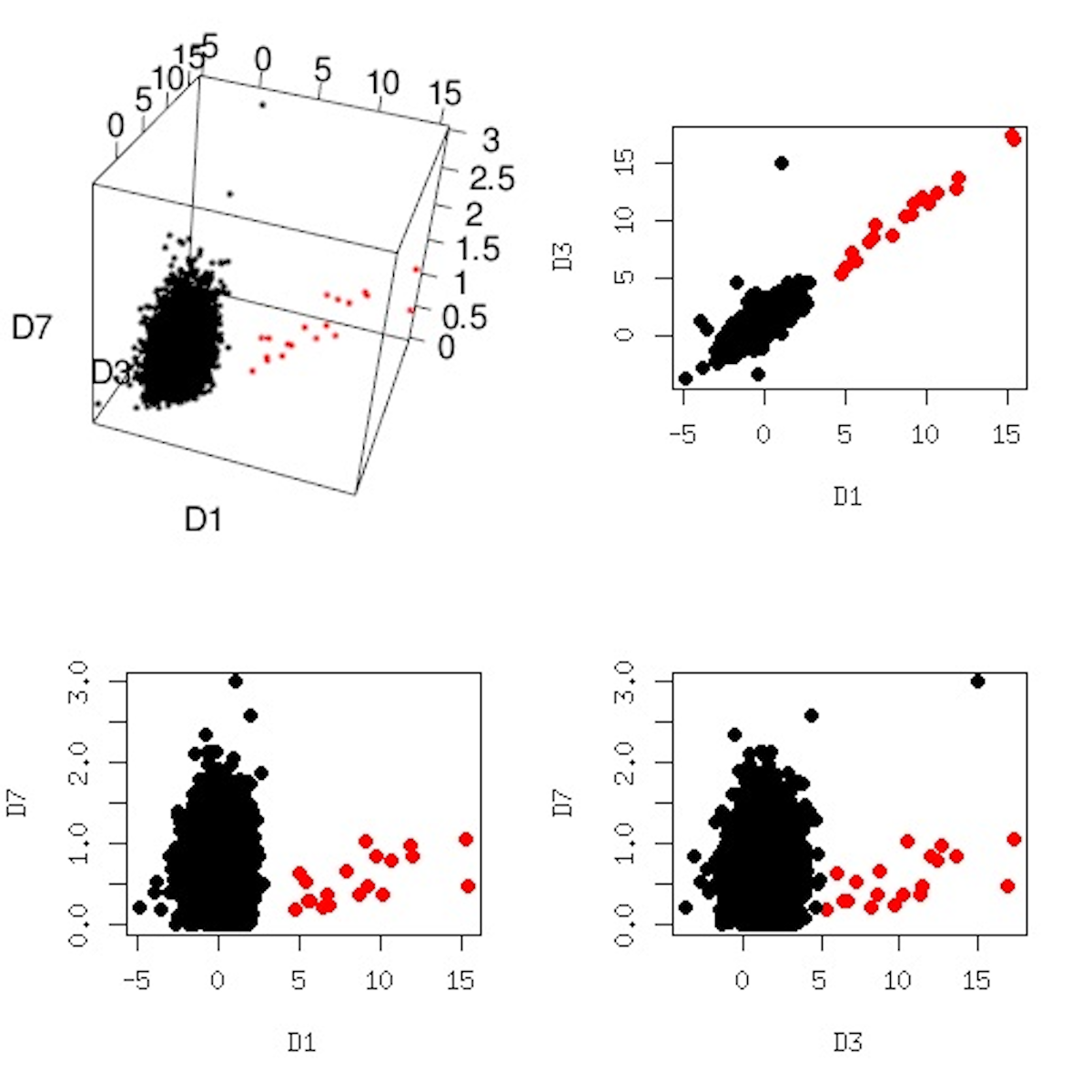

Supplement: S3 Fig — Features D1, D3 and D7 were employed in this visualization and represented as X-axis, Y-axis and Z-axis respectively. Dark solid points illustrate the negative samples and red solid points the positive samples. (TIFF) [file pone.0207840.s003.tiff]
